# Development of Magnetic Nanobeads Modified by Artificial Fluorescent Peptides for the Highly Sensitive and Selective Analysis of Oxytocin

**DOI:** 10.3390/s20205956

**Published:** 2020-10-21

**Authors:** Yoshio Suzuki

**Affiliations:** Health and Medical Research Institute, National Institute of Advanced Industrial Science and Technology (AIST), 1-1-1 Higashi, Tsukuba, Ibaraki 305-8566, Japan; suzuki-yoshio@aist.go.jp; Tel.: +81-29-861-6122

**Keywords:** fluorescence, oxytocin, peptide, molecular probes, magnetic beads, nanosensor

## Abstract

We describe two novel fluorescent peptides (compounds 1 and 2) targeting oxytocin with a boron-dipyrromethenyl group as the fluorophore bound to an artificial peptide based on the oxytocin receptor, and their application for the analysis of oxytocin levels in human serum using nanometer-sized magnetic beads modified by fluorescent peptides (FMB-1 and FMB-2). Under the optimized experimental protocols, FMB-1 and FMB-2 emitted low levels of fluorescence but emitted much higher levels of fluorescence when associated with oxytocin. The detection limit of oxytocin by FMB-2 was 0.4 pM, which is approximately 37.5 times higher than that of conventional methods, such as ELISA. Using these fluorescent sensors, oxytocin was specifically detected over a wide linear range with high sensitivity, good reusability, stability, precision, and reproducibility. This fluorescent sensor-based detection system thus enabled the measurement of oxytocin levels in human serum, which has widespread applications for oxytocin assays across varied research fields.

## 1. Introduction

Oxytocin is a pituitary peptide produced in the paraventricular nucleus of the hypothalamus and stored in the posterior pituitary [1,2,3,4]. Oxytocin consists of a semi-flexible amidated C-terminus and nine amino acids with disulfide bonds. Oxytocin was once thought to only be present in the smooth muscle reproductive physiology of females. However, in recent years, oxytocin has been implicated in neuropsychiatric disorders, such as autism spectrum disorder, and social/sexual behavior as a neurotransmitter, as well as in male reproductive physiology [5,6,7,8]. The efficacy of oxytocin administered therapeutically depends on its dosage, and human studies on autism spectrum disorder depend on oxytocin efficacy [9,10,11,12].

To date, various biosensing methods have been developed for the detection of oxytocin, mostly by ELISA and LC–MS [13,14,15,16]. However, these methods have several limitations, including low throughput, high operation costs, the requirement for expensive instrumentation, and for trained personnel. Therefore, it is essential to develop efficient and rapid methods that can be used to selectively measure and continuously detect changes in oxytocin levels in the body.

Currently, sensors based on nanomaterials have been developed for the detection of various chemical substances by combining them with optical analytical methods such as fluorescence spectroscopy [17,18,19,20]. To improve the performance of these sensors, it is necessary to develop fluorescent probes that exhibit a spectral response upon binding to ions and neutral organic or inorganic molecules. This will enable the measurement of changes in free guest ions or changes in the concentration of bio- and environmental molecules with high sensitivity and selectivity [21,22,23,24,25,26,27]. This approach generally has a high throughput and represents a highly sensitive and selective method for the analysis of target molecules.

To design fluorescent probes for use in an oxytocin assay, we assumed a weak-to-strong performance enhancement of the fluorescence signal upon binding to oxytocin. In addition, we assumed that the introduction of a selective recognition site to the probe would not lead to losses in optical properties and stability. This allowed for the development of probes with a reduced background, high sensitivity, high selectivity, and reduced interference from other substances.

We previously developed a highly sensitive fluorescent reagent for oxytocin detection [28]. However, the photostability of this reagent was poor due to the poor lifetime of the cyanopyranyl moiety in the fluorescent probe, which may lead to incorrect results and does not allow the detection of physiological levels of oxytocin in long-term measurements.

Therefore, in this study, we aimed to develop an improved method for oxytocin detection that makes use of the properties of fluorescent molecular probes. The probes were constructed using the boron-dipyrromethenyl group as a fluorescent unit because of its interesting spectral properties, such as high quantum yields, large extinction coefficients [29,30], high photostability, and an oligopeptide processor for both oxytocin and spacer binding sites. The design of the amino acid sequences was based on the oxytocin receptor [31], with compound 1 being based on our previously reported compound [28], and compound 2 having a novel design. Compounds 1 and 2 were successfully synthesized and their chemical structures were investigated using high-performance liquid chromatography (HPLC) and mass spectrometry. We developed nanometer-sized fluorescent magnetic beads (FMB-1 and FMB-2) to detect oxytocin based on the enhanced fluorescence-changing activity of the two compounds. We assessed the specificity, sensitivity, stability, reusability, and oxytocin-detection capability of our novel oxytocin assay in human serum. Our novel oxytocin assay would enable long-term longitudinal measurements of oxytocin at physiological levels in plasma samples, supporting the administration of precise doses of therapeutic oxytocin in several important human diseases, as well as an accurate monitoring of oxytocin levels.

## 2. Materials and Methods

### 2.1. Chemicals and Reagents

All the chemicals used in this study were of analytical grade and were purchased from Tokyo Chemical Industry (TCI, Tokyo, Japan), Wako Pure Chemical Industries, Ltd. (Osaka, Japan), and GE Healthcare (Chicago, IL, USA). Magnetic beads were purchased from Tamagawa Seiki Co., Ltd. (Nagano, Japan). Fluorescence spectra were recorded at 25 °C using a JASCO FP-6500 fluorophotometer and FP-8300 fluorophotometer.

Control serum was purchased from Wako Pure Chemical Industries, Ltd. (Osaka, Japan). This reagent contained IgG (1005 ± 100 mg/dL), IgA (193 ± 19 mg/dL), IgM (77 ± 7 mg/dL), C3 (110 ± 11 mg/dL), C4 (19.4 ± 1.9 mg/dL), CRP (0.79 ± 0.07 mg/dL), RF (23 ± 3 IU/mL), and ASO (90 ± 13 IU/mL).

### 2.2. Synthesis of Fluorescent Peptides for the Detection of Oxytocin

The synthesis of *N*-(5-(5,5-difluoro-1,3,7,9-tetramethyl-*5H*-4λ^4^,5λ^4^-dipyrrolo [1,2-c:2’,1’-f] [1,3,2]diazaborinin-10-yl)-2-hydroxybenzyl)-*N*-methylglycine from 4-hydroxybenzaldehyde was performed according to our previously published method [32]. The synthesis of the fluorescent peptides (compounds 1 and 2) is detailed below.

Each peptide (5.0 mg) was esterified at the C-terminal by a methyl group, then added with 0.9 mg 1-ethyl-3-(3-dimethylaminopropyl)-carbodiimide hydrochloride and 0.6 mg *N*-ethyldiisopropylamine to a solution of *N*-(5-(5,5-difluoro-1,3,7,9-tetramethyl-*5H*-4λ^4^,5λ^4^-dipyrrolo [1,2-c:2’,1’-f] [1,3,2]diazaborinin-10-yl)-2-hydroxybenzyl)-*N*-methylglycine in 5.0 mL of *N,N*-dimethylformamide, and stirred for 12 h at 25 °C under nitrogen atmosphere. After removing the solvent, the residue was purified using HPLC (ODS column; pump A, 0.06% TFA in water; pump B, 0.05% TFA in CH_3_CN).

The esterified fluorescent peptides were dissolved in 0.1 N NaOH and stirred for 12 h at 25 °C. After removal of the solvent, the residue was purified by HPLC (ODS column; pump A, 0.06% TFA in water; pump B, 0.05% TFA in CH_3_CN). The yield of the process and the results of mass spectrometry were as follows:

Compound 1: Yield 87%; HPLC purity 96.0%; MALDI-TOF MS (+): [M + H]^+^ = 3512.89 (calculated for 3512.88).

Compound 2: Yield 85%; HPLC purity 95.7%; MALDI-TOF MS (+): [M + H]^+^ = 6077.81 (calculated for 6077.82).

### 2.3. Synthesis of Fluorescent Magnetic Beads (FMB-1 and FMB-2)

Immobilization of the fluorescent peptides onto magnetic beads, detection of oxytocin using fluorescent peptides, and the detection of oxytocin using fluorescent peptides immobilized on magnetic beads have been described previously [28]. The optimal experimental conditions for the immobilization of fluorescent peptides onto magnetic beads were investigated by analyzing the responses of fluorescent magnetic beads in the presence of oxytocin at different reaction times. For each peptide, 2.5 mg of magnetic beads in 500 μL of DMF was added to a solution containing different concentrations of compound 1 or 2 (0–10 μM), esterified by 1-(3-Dimethylaminopropyl)-3-ethylcarbodiimide Hydrochlorideand *N*-Hydroxysuccinimide. The reaction was then monitored by varying the reaction time (0, 1, 2, 5, 10, 15, 20, and 24 h). The optical change in the fluorescence emitted by the fluorescent magnetic beads (FMB-1 and FMB-2) was investigated by the addition of 0.5 nM oxytocin. We determined the optimal experimental conditions based on the combination of reaction time and peptide concentration that gave the highest fluorescence reading in the presence of oxytocin.

### 2.4. Performance Evaluation of Fluorescent Magnetic Beads in Oxytocin Level Measurement

FMB-1 or FMB-2 were added to HEPES buffer solution (pH 7.0) to obtain a final concentration of 80 μg/mL. After the addition of 100 μL oxytocin (0–1000 pM) to 100 μL of FMB-1 or FMB-2, the reaction mixture was incubated for 50 min. After concentrating FMB-1 or FMB-2 using a magnet, FMB-1 or FMB-2 were washed briefly, and the fluorescence spectra were recorded.

Selectivity was measured by comparing the response of 100 μL of FMB-1 or FMB-2 after the addition of 100 μL of oxytocin or other substances, including albumin, globulin, sodium, potassium, calcium, magnesium, glucose, lactic acid, creatine, creatinine to control serum. The reaction mixture was incubated for 50 min. After concentrating FMB-1 or FMB-2 using a magnet, the beads were washed briefly, and fluorescence spectra were recorded.

The reusability of the beads was tested by performing multiple cycles of the reaction between FMB-1 (or FMB-2) and oxytocin and brief washing with aqueous NaOH to remove oxytocin from the magnetic beads. After completing the assay, the fluorescent magnetic beads were reused to measure the residual activity.

The stability of FMB-1 and FMB-2 was analyzed by incubating the beads at 4 °C and calculating their residual activity by measuring the fluorescence intensity over an extended time period.

## 3. Results and Discussion

**Figure 1:**
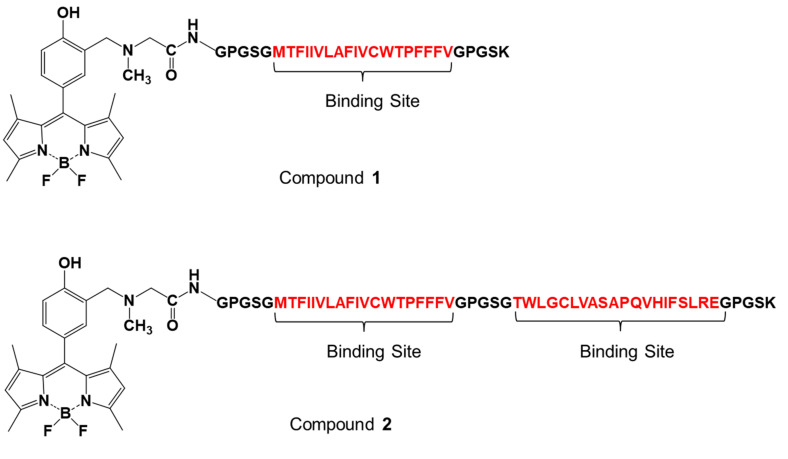
Chemical structures and peptide sequences of artificial fluorescent peptides for the measurement of oxytocin levels (designated as compound **1** and compound **2**).

**Figure 2:**
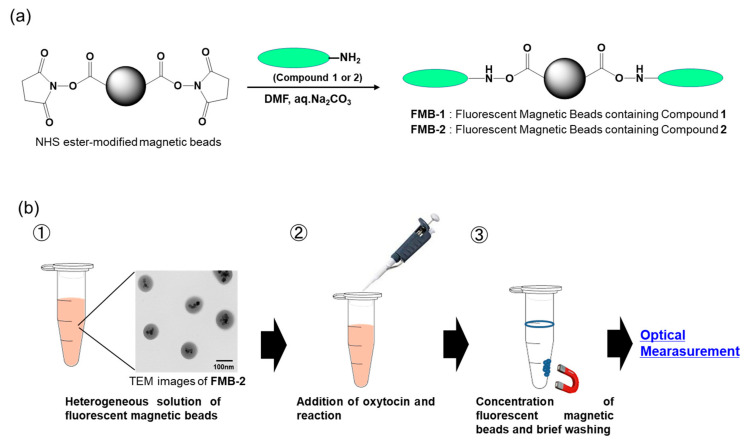
Schematic representation of the immobilization of fluorescent peptides onto the surface of fluorescent magnetic beads (designated FMB-1 and FMB-2) (**a**) and the experimental procedure for the measurement of oxytocin levels using these beads (**b**).

Artificial fluorescent peptides, designated as compound 1 and compound 2 (Figure 1), were immobilized on the surface of magnetic beads via the reaction of an amino group at the side chain of lysine at the C-terminus of the peptides with NHS ester groups on the magnetic beads to create FMB-1 and FMB-2, according to the workflow shown in Figure 2a. The synthesis of FMB-1 and FMB-2 was carried out to measure oxytocin levels using a fluorescent assay. Fluorescence intensities of FMB-1 and FMB-2 were recorded before and after the addition of different concentrations of oxytocin. The workflow of the experimental procedure for the measurement of oxytocin levels is shown in Figure 2b.

Oxytocin concentrations ranging between 0 and 1000 pM were added to a suspension of fluorescent magnetic beads and incubated for 50 min. After magnet-driven concentration and a brief wash, the response of the fluorescent magnetic beads before and after the addition of oxytocin was monitored. Typical absorption spectra and the relationships between the concentration of FMB-1 or FMB-2 and the measured absorbance are shown in Supplementary Figures S5 and S6. The absorbances of FMB-1 and FMB-2 at 500 nm were 0.14 and 0.13, respectively, and the low concentrations of FMB-1 and FMB-2, in combination with the excitation wavelength of 490 nm, which was set at 10 nm below the absorption maximum, avoided inner filter effects.

**Figure 3:**
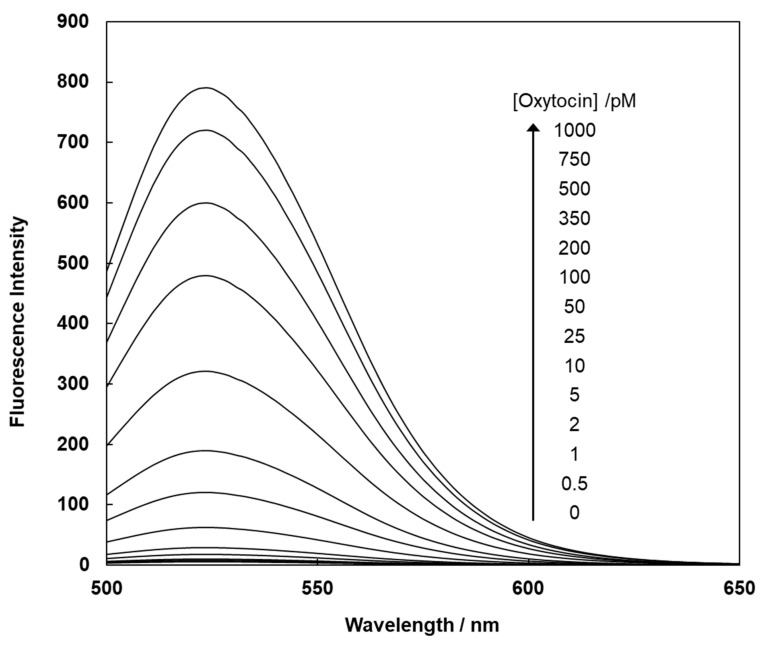
Fluorescence spectra of FMB-2 before and after addition of varying concentrations of oxytocin. [FMB-2] = 40 μg/mL; [Oxytocin] = 0~1000 pM; solvent = 20.0 mM HEPES buffer (pH 7.0); excitation wavelength = 490 nm.

The magnetic beads exhibited a very weak fluorescence emission in the absence of oxytocin; however, a strong fluorescent signal was observed after the addition of oxytocin (Figure 3). The fluorescence intensity of FMB-2 at 525 nm increased from 6.0 to approximately 789.8 following the addition of oxytocin, corresponding to a 131.5-fold increase in fluorescent signal. Similar results were observed for the interaction between FMB-1 and oxytocin (Supplementary Figure S7).

**Figure 4:**
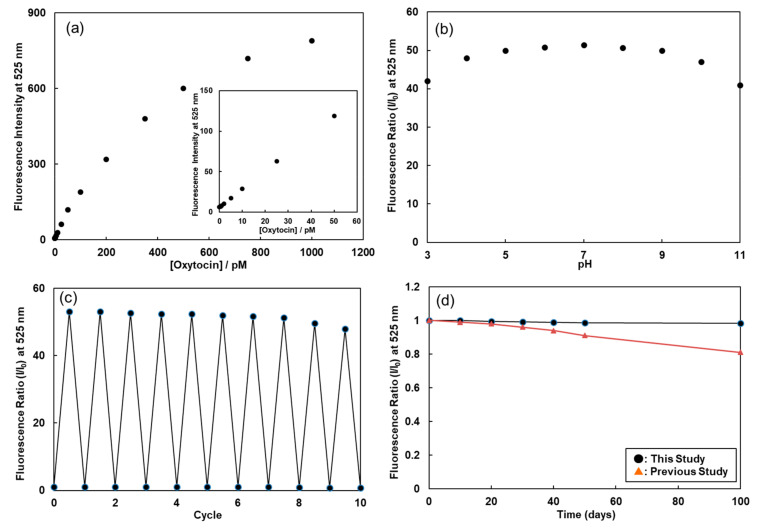
Fluorescence intensities (at 525 nm) of FMB-2 after the addition of different concentrations of oxytocin (**a**), the fluorescence ratio of FMB-2 after the addition of oxytocin in buffer at different pH (**b**), the reusability (**c**), and storage stability (**d**) of fluorescent magnetic beads. [FMB-2] = 40 μg/mL; excitation wavelength, 490 nm; storage temperature, 4 °C.

The emission intensities of FMB-2 at 525 nm were plotted as a function of oxytocin concentration. Figure 4a shows a typical calibration graph showing the dependence of the intensities on the oxytocin concentration under optimal experimental conditions. This plot exhibits a sigmoidal curve relationship between the emission intensities of FMB-2 and the fluorescence intensity—up to 1000 pM in concentration. The calibration graph of FMB-1 indicated increasing fluorescence as a function of oxytocin concentration, with fluorescence intensities five times lower than those of FMB-2 (Supplementary Figure S8).

The detection limit and detectable range of FMB-2 were 0.4 pM (signal-to-noise ratio of 3.0) and 0.4~1000 pM for oxytocin, whereas those of FMB-1 were 2.1 pM (signal-to-noise ratio of 3.0) and 2.0~500 pM. The fluorescent peptides (compound 1 and compound 2) immobilized on these magnetic beads have amino acid sequences that are based on the oxytocin receptor (Figure 1), and these amino acid sequences act as binding sites for oxytocin. Compound 1 has one binding site, whereas compound 2 has two. This structural difference contributes to the observed increase in sensitivity for FMB-2 compared to FMB-1 [28]. The amino acid sequence of the peptide bound to the magnetic beads therefore greatly affects the sensitivity of fluorescent sensors.

The response of FMB-2 to various contaminants, such as inorganic salts, proteins, and other foreign substances which may be present in biological samples, was tested to investigate the specificity of the fluorescent magnetic-bead based oxytocin assay. The fluorescence ratios of FMB-2 at 525 nm were monitored before and after the addition of the interfering substances (Supplementary Figure S9). A large increase in the fluorescence ratio was observed when oxytocin was added, with a fluorescence ratio at 525 nm of 46. In contrast, there was no significant increase in the fluorescence ratio after the addition of the interfering substances. Fluorescent magnetic beads mixed in control serum showed a large increase in fluorescence after the addition of oxytocin, and the fluorescence ratio of fluorescent magnetic beads after the addition of oxytocin in the control serum was similar to that observed in buffer-dissolved oxytocin. We can therefore conclude that FMB-2 was able to detect oxytocin with high selectivity in the presence of foreign substances, including in a human serum background matrix. The binding sites of FMB-2 were based on the oxytocin receptor, resulting in stable complex formation between FMB-2 and oxytocin, and causing a large increase in fluorescence intensity compared to that of any non-specific binding with contaminants.

To investigate the pH dependence of the reaction time between oxytocin and FMB-2, the fluorescence intensities of FMB-2 mixed with 0.5 nM oxytocin were measured at pH values ranging from 3 to 11. Our fluorescent magnetic beads exhibited inhibition at high pH (10–11) and low pH (3–4). However, the fluorescence intensities were nearly unchanged when the pH was in the range of 5 to 9, which corresponds to the physiological pH of the human body (Figure 4b). Fluorescent magnetic beads therefore satisfy the experimental conditions for the analysis of physiological processes in the human body, suggesting that our novel assay is applicable in the clinical setting for the measurement of oxytocin in human plasma samples.

We next examined the reusability of the fluorescent magnetic beads for the detection of oxytocin. Any clinical assay would greatly benefit from the reusability of consumables, which decreases costs as well as reducing the environmental impact of producing and disposing these consumables. Reusability was measured after cycles of the reaction were performed using the magnetic beads and oxytocin, with brief washes between each cycle to remove unreacted oxytocin. After the release of oxytocin from the surface of the magnetic beads by the addition of NaOH(aq) and washing, we monitored the initial fluorescence intensity, as shown in Figure 4c. The fluorescent magnetic beads retained their initial fluorescence ratio for up to eight cycles, with an approximate 5% decrease from the initial fluorescence intensity. This demonstrates the excellent reusability of fluorescent magnetic beads for the measurement of oxytocin levels using our assay.

The stability of FMB-2 is an important factor for the long-term measurement of oxytocin. The fluorescent magnetic beads were stored in 1.5-mL tubes at 4 °C, and the fluorescence ratio was monitored at intervals of 10 days. FMB-2 preserved over 98% of its initial fluorescence intensity for up to 5 months (Figure 4d). However, as reported previously, the fluorescence ratio of fluorescent magnetic beads decreased slowly with time. The fluorescent magnetic beads developed for the present study are very stable because of their durable fluorophore, and therefore have long-lasting activity toward oxytocin, thus extending their shelf-life in clinical settings.

Finally, the fluorescent magnetic beads were applied to measure the levels of oxytocin in human serum that had been spiked with oxytocin representative of physiological oxytocin levels. FMB-2 exhibited a rapid increase in fluorescence intensity with the addition of human serum samples. We quantified the oxytocin concentrations in the human serum with CV values of 2.7% and recovery rates of approximately 100.9%. The data are summarized in Table 1. This indicates that the serum matrix did not interfere with the reaction between the fluorescent magnetic beads and oxytocin, and that this sensor could therefore provide excellent reproducibility and reliability for the measurement of oxytocin levels in human blood samples.

We compared the characteristics of our novel magnetic bead-based assay for oxytocin with those of existing methods (Table 2). The enzyme-linked immunosorbent assay (ELISA) is generally considered to be the gold standard when it comes to the measurement of proteins in biofluids. However, the ELISA is slow and complex, and the analytical reagents are expensive and not reusable. Moreover, oxytocin released from platelets in the blood sample can result in an incorrect readout when using ELISA. For these reasons, we propose that our magnetic bead-based oxytocin assay is superior to ELISA for the measurement of oxytocin in serum samples. Our method is more sensitive, and its operation time is much shorter than ELISA’s. Moreover, the magnetic beads are reusable, have a wide detection range, and are highly selective for oxytocin, which allows the successful detection of oxytocin in human serum. The oxytocin assay using FMB-2 is a highly sensitive, selective, and rapid method for the detection of oxytocin, and can be widely used as a high-throughput method for the measurement of oxytocin levels in human biofluids.

## 4. Conclusions

In the present study, we developed a novel fluorescent peptide for the detection of oxytocin, and nanometer-sized magnetic beads containing these peptides were synthesized using a conventional synthetic method. Changes in fluorescence indicate the successful binding of the target oxytocin with the fluorescent peptide. We observed large changes in fluorescence in the presence of oxytocin, indicating a high sensitivity and selectivity, as well as demonstrating good reusability and durability of these magnetic beads. These parameters contribute to the detection of oxytocin in human serum samples with high precision. This sensing system is easy to use and offers rapid and highly sensitive detection based on the unique properties of fluorescence enhancement. We are currently investigating other possible molecular probes for this system, which could be used to detect a range of biological substances that may be useful in research and medical fields.

## Figures and Tables

**Table 1:** Detection of oxytocin using FMB-2 in human serum.

Added Oxytocin (pM)	Expected (pM)	Measured (pM) ^a^	CV (%) ^b^	Recovery (%) ^c^
10.00	10.00	10.09	2.7	100.9

^a^ The average value of five independent measurements; ^b^ Coefficient of variation; ^c^ (Measured value) ÷ (Expected value) × 100.

**Table 2:** Comparison of the methods of this study with those of other oxytocin detection studies.

Assays	Detection Limit (pM)	Detectable Range (pM)	Operation Time (Hours)	Stability	Reference
**FMB-1**	2.0	2.0~500	1.0	○	-
**FMB-2**	0.4	0.4~1000	1.0	○	-
Our previous study	2.0	2.0~500	1.0	×	[28]
ELISA	15.0	15.6~1000	12.0	-	[33]

## Supplementary Materials

The following are available online at https://www.mdpi.com/1424-8220/20/20/5956/s1, Supplementary Figure S1: MALDI-TOF MS spectra of compound 1; Supplementary Figure S2: HPLC chromatogram of compound 1; Supplementary Figure S3: MALDI-TOF MS spectra of compound 2; Supplementary Figure S4: HPLC chromatogram of compound 2; Supplementary Figure S5: Absorption spectrum of FMB-1 at 40 μg/mL (a), and the relationship between the absorbance at 500 nm and the concentration of FMB-1 (b); Supplementary Figure S6: Absorption spectrum of FMB-2 at 40 μg/mL (a), and the relationship between the absorbance at 500 nm and the concentration of FMB-2 (b); Supplementary Figure S7: Fluorescence spectra of FMB-1 before and after the addition of different concentrations of oxytocin; Supplementary Figure S8: Fluorescence intensity (at 525 nm) of FMB-1 after the addition of different concentrations of oxytocin; Supplementary Figure S9: Fluorescence intensity of FMB-2 at 525 nm before and after the addition of oxytocin or potential contaminants.
