# Supplementary material for: Development of Magnetic Nanobeads Modified by Artificial Fluorescent Peptides for the Highly Sensitive and Selective Analysis of Oxytocin
Source: Sensors (Basel). 2020 Oct 21;20(20):5956. doi: 10.3390/s20205956 (PMC7588987; doi:10.3390/s20205956)
Supplement: Supplementary file 1 [file sensors-20-05956-s001.pdf]

sensors

MDPI

Supplementary Materials

Development of Magnetic Nanobeads Modified by Artificial Fluorescent Peptides for the Highly Sensitive and Selective Analysis of Oxytocin

Yoshio Suzuki

Health and Medical Research Institute, National Institute of Advanced Industrial Science and Technology (AIST), 1-1-1 Higashi, Tsukuba, Ibaraki 305-8566, Japan; suzuki-yoshio@aist.go.jp; Tel.: +81-29-861-6122

Contents:

Supplementary Figure S1: MALDI-TOF MS spectra of compound **1**.

Supplementary Figure S2: HPLC chromatogram of compound **1**. Experimental conditions are as follows; Flow rate: 1.0 mL/min, Mobile phase: Pump A; 0.06% TFA in 100% water (v/v) Pump B: 0.05% TFA in 100% acetonitrile (v/v).

Supplementary Figure S3: MALDI-TOF MS spectra of compound **2**.

Supplementary Figure S4: HPLC chromatogram of compound **2**. Experimental conditions are as follows; Flow rate: 2.0 mL/min, Mobile phase: Pump A: 0.06% TFA in 100% water (v/v) Pump B: 0.05% TFA in 100% acetonitrile (v/v).

Supplementary Figure S5: Absorption spectrum of **FMB-1** at 40 μg/mL (a), and the relationship between the absorbance at 500 nm and the concentration of **FMB-1** (b). [**FMB-1**] = 0 ~ 200 μg/mL; solvent = 20.0 mM HEPES buffer (pH 7.0).

Supplementary Figure S6: Absorption spectrum of **FMB-2** at 40 μg/mL (a), and the relationship between the absorbance at 500 nm and the concentration of **FMB-2** (b). [**FMB-2**] = 0~200 μg/mL; solvent = 20.0 mM HEPES buffer (pH 7.0).

Supplementary Figure S7: Fluorescence spectra of **FMB-1** before and after the addition of different concentrations of oxytocin. [**FMB-1**] = 40 μg/mL; [Oxytocin] = 0~1000 pM; solvent = 20.0 mM HEPES buffer (pH 7.0); excitation wavelength = 490 nm.

Supplementary Figure S8: Fluorescence intensity (at 525 nm) of **FMB-1** after the addition of different concentrations of oxytocin. [**FMB-1**] = 40 μg/mL; excitation wavelength, 490 nm.

Supplementary Figure S9: Fluorescence intensity of **FMB-2** at 525 nm before and after the addition of oxytocin or potential contaminants. [**FMB-2**] = 40 μg/mL; [Oxytocin] = 500 pM; [albumin] = 5.0 g/dL; [globulin] = 5.0 g/dL; [sodium] = 190 mg/dL; [potassium] = 190 mg/dL; [calcium] = 190 mg/dL; [magnesium] = 190 mg/dL; [glucose] = 100 mg/dL; [lactic acid] = 15 mg/dL; [creatine] = 3.9 mg/dL; [creatinine] = 0.9 mg/dL; excitation wavelength = 490 nm. I is the fluorescence intensity of **FMB-2** at 525 nm before and after the addition of the potential contaminants, and I_0 is the fluorescence intensity of **FMB-2** at 525 nm.

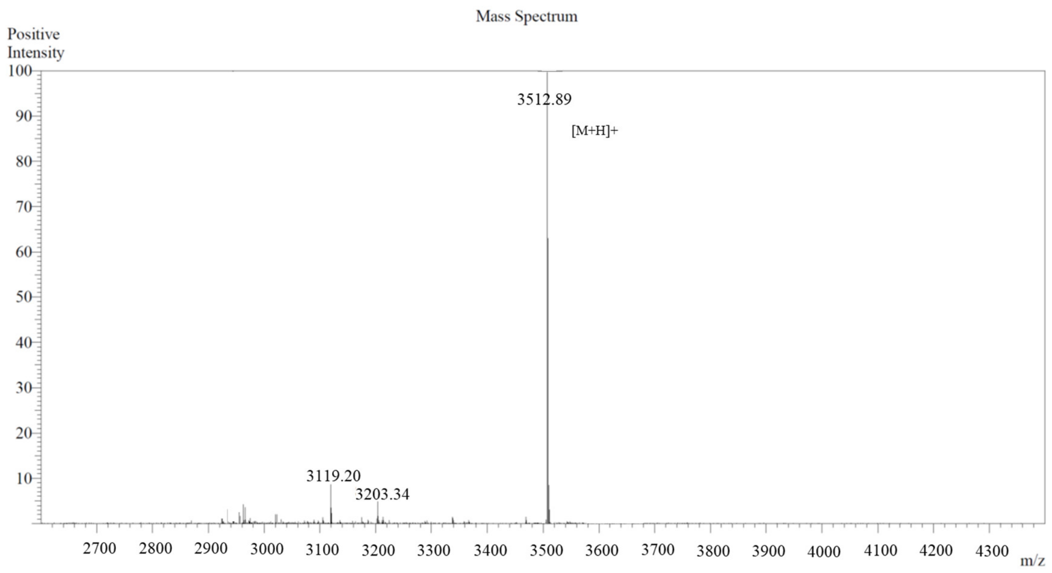

Figure S1. MALDI-TOF MS spectra of compound **1**.

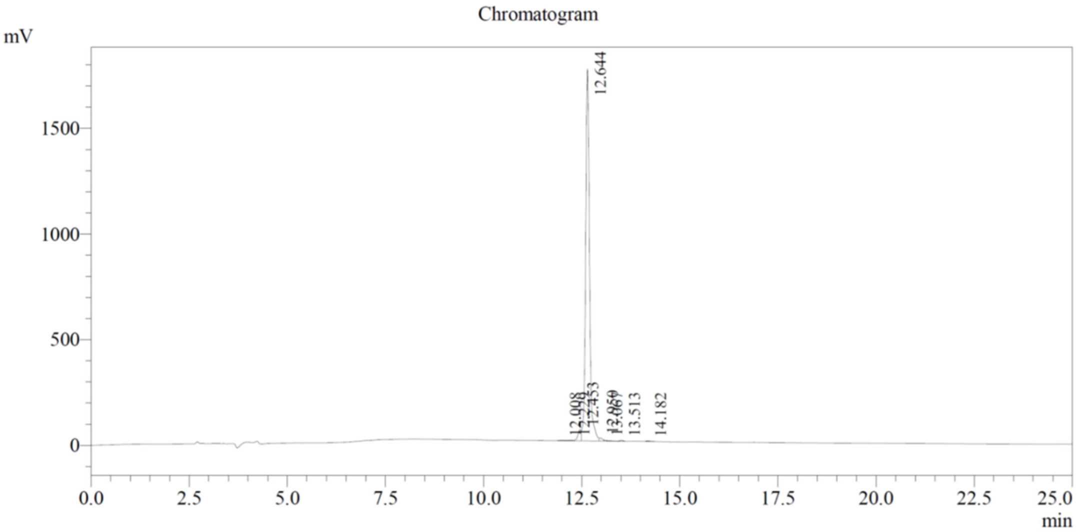

.

Figure S2. HPLC chromatogram of compound **1**. Experimental conditions are as follows: Flow rate, 1.0 mL/min; Mobile phase: Pump A, 0.06% TFA in 100% water (v/v); Pump B, 0.05% TFA in 100% acetonitrile (v/v).

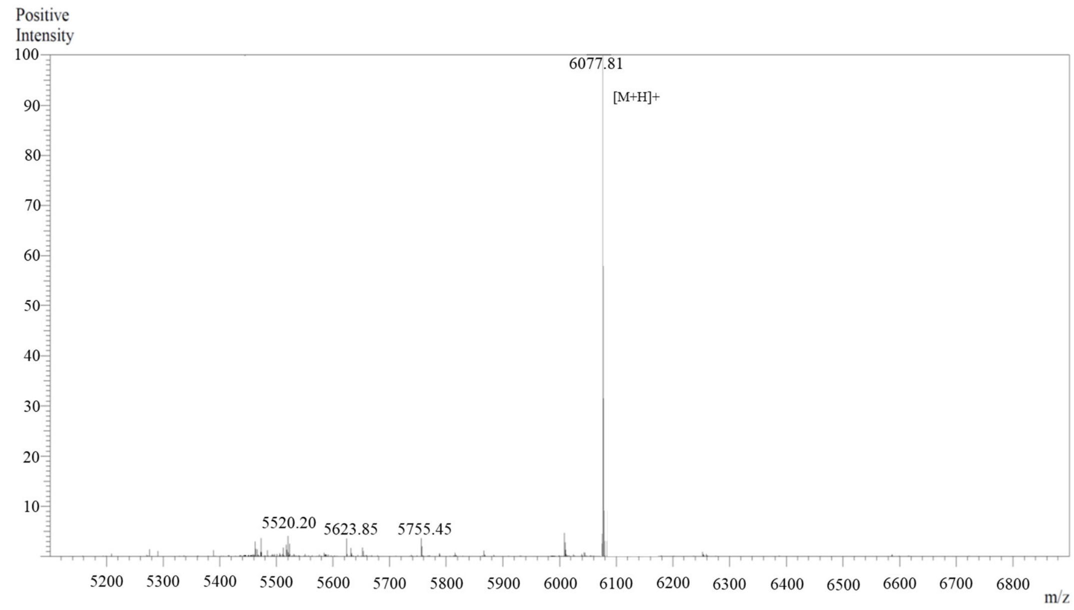

Figure S3. MALDI-TOF MS spectra of compound **2**.

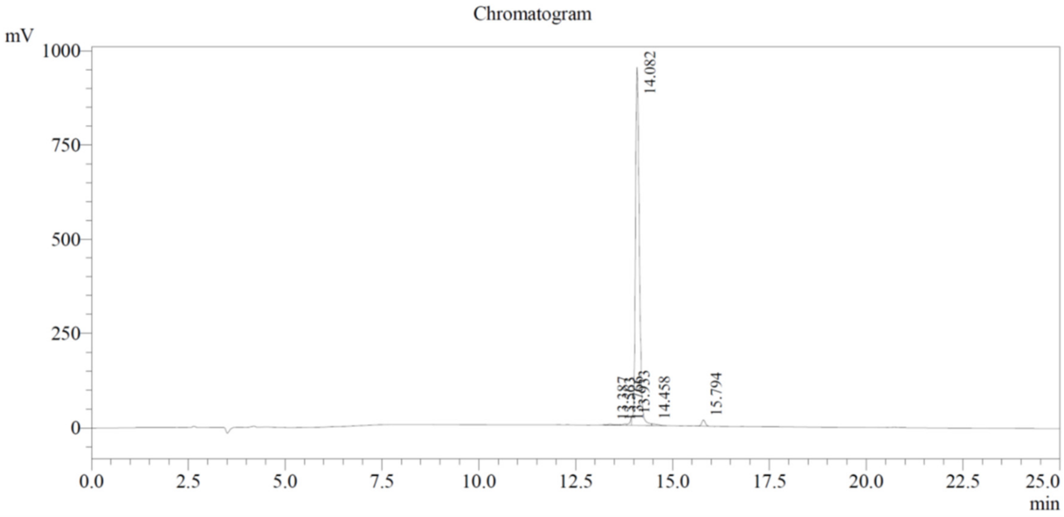

Figure S4. HPLC chromatogram of compound **2**. Experimental conditions are as follows: Flow rate, 2.0 mL/min; Mobile phase: Pump A, 0.06% TFA in 100% water (v/v), Pump B, 0.05% TFA in 100% acetonitrile (v/v).

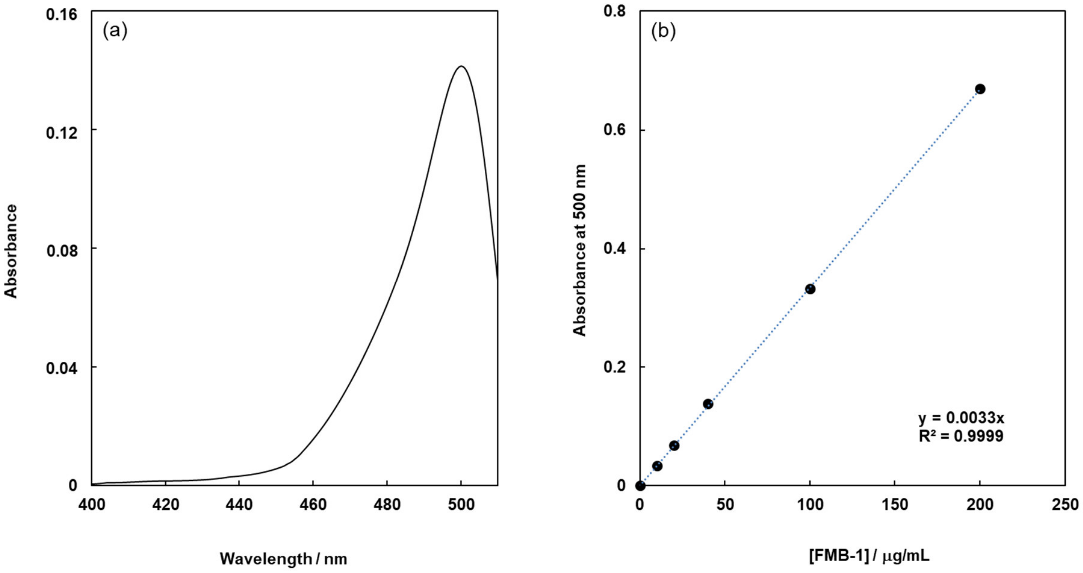

Figure S5. Absorption spectrum of **FMB-1** whose concentration was 40 μg/mL (a), and the relationship between the absorbance at 500 nm and the concentration of **FMB-1** (b). [**FMB-1**] = 0~200 μg/mL; solvent = 20.0 mM HEPES buffer (pH 7.0).

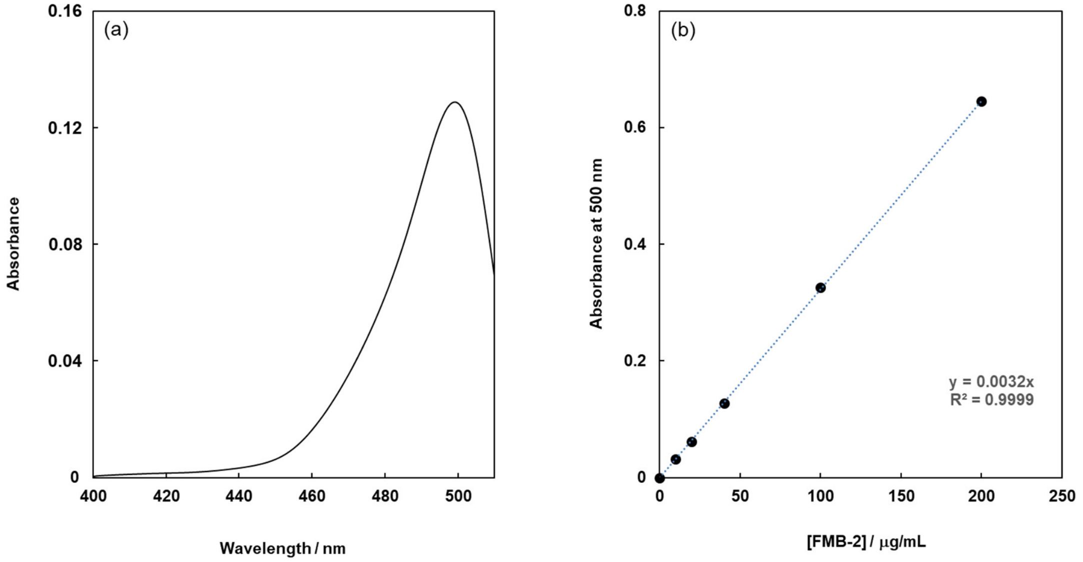

Figure S6. Absorption spectrum of **FMB-2** whose concentration was 40 μg/mL (a), and the relationship between the absorbance at 500 nm and the concentration of **FMB-2** (b). [**FMB-2**] = 0~200 μg/mL; solvent = 20.0 mM HEPES buffer (pH 7.0).

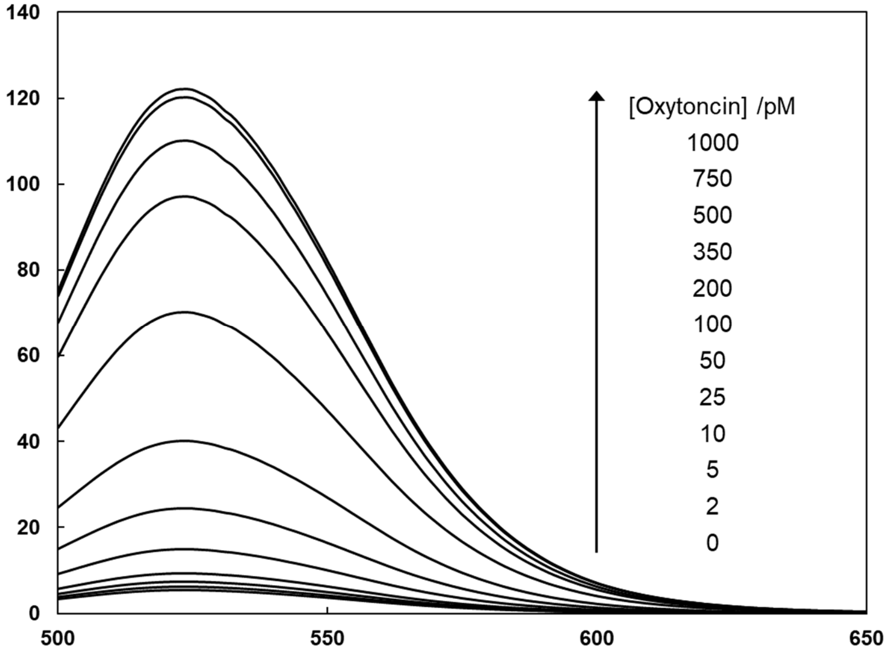

Figure S7. Fluorescence spectra of **FMB-1** before and after addition of varying concentrations of oxytocin. [**FMB-1**] = 40 µg/mL; [Oxytocin] = 0~1000 pM; solvent = 20.0 mM HEPES buffer (pH 7.0); excitation wavelength = 490 nm.

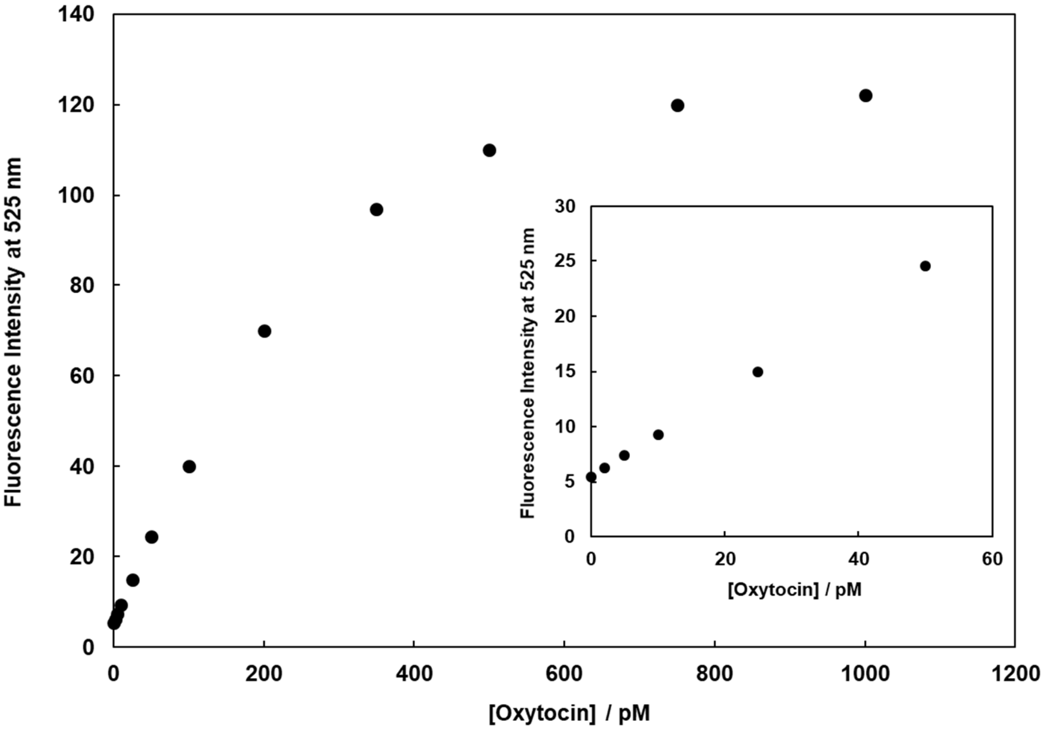

Figure S8. Fluorescence intensities (at 525 nm) of **FMB-1** after the addition of various concentrations of oxytocin. [**FMB-1**] = 40 µg/mL; excitation wavelength, 490 nm.

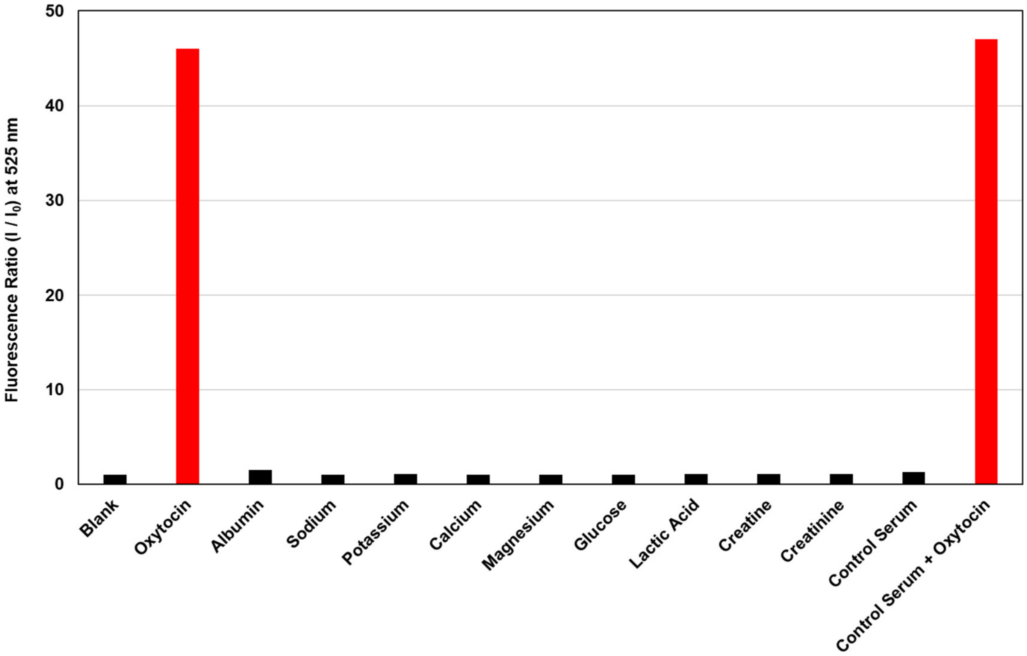

Figure S9. Fluorescence intensity of **FMB-2** at 525 nm before and after the addition of oxytocin and various foreign substances. [**FMB-2**] = 40 µg/mL; [Oxytocin] = 500 pM; [albumin] = 5.0 g/dL; [globulin] = 5.0 g/dL; [sodium] = 190 mg/dL; [potassium] = 190 mg/dL; [calcium] = 190 mg/dL; [magnesium] = 190 mg/dL; [glucose] = 100 mg/dL; [lactic acid] = 15 mg/dL; [creatine] = 3.9 mg/dL; [creatinine] = 0.9 mg/dL; excitation wavelength = 490 nm. I is the fluorescence intensity of **FMB-2** at 525 nm before and after the addition of various compounds, and I_0 is the fluorescence intensity of **FMB-2** at 525 nm.
